# Risk factors for stroke-related functional disability and mortality at Felege Hiwot Referral Hospital, Ethiopia

**DOI:** 10.1186/s12883-023-03444-8

**Published:** 2023-10-31

**Authors:** Tegenu Tento, Abraham Kume, Sebisibe Kumaso

**Affiliations:** 1 Department of Statistics, College of Natural and Computational Sciences, Jinka University, Jinka, Ethiopia; 2 Health Monitoring and Evaluation Department, Alle Special Woreda, Kolango, Ethiopia

**Keywords:** Hazard ratio, Instantaneous risk, Transition

## Abstract

**Background:**

Stroke is one of the top causes of functional disability around the world. The main objective was to identify stroke-related functional outcomes and risk factors. A good functional outcome is defined as the absence of problems secondary to the stroke event, a poor functional outcome as the presence of complications, and mortality as the existence of complications.

**Method:**

A retrospective cohort analysis was used to observe factors in 298 eligible adult (18 or older) stroke patients who attend outpatient clinics every three months at Felege Hiwot Referral Hospital between September 2019 and August 2021 to predict outcomes.

**Result:**

The likelihood of dying from a poor outcome was 9%, and the likelihood of recovering was 24%. The average time spent on good and poor outcomes for different levels of independent variables varies according to their risk. During the first three years of follow-up, the instantaneous risk with a 95% confidence interval of transitioning from good to poor outcome in the women, aged 60 or older, with hypertension, atrial fibrillation, and hemorrhage stroke versus men stroke patients, aged 18 to 59, without hypertension, atrial fibrillation, and ischemic stroke were 1.54 (1.10, 2.15), 1.73 (1.19, 2.52), 2.34 (1.55, 3.53), 2.74 (1.64, 4.56), and 1.52 (1.10, 2.19) respectively. The hazard ratio of transitioning from poor outcome to death for patients with diabetes mellitus and atrial fibrillation versus those without diabetes mellitus and atrial fibrillation was estimated to be 1.95 (1.10, 3.46) and 3.39 (1.67, 6.89), respectively.

**Conclusion:**

Women over 60 with hypertension, atrial fibrillation, and hemorrhagic stroke were more likely to progress from a good to a poor outcome. Diabetes and atrial fibrillation were also risk factors for progressing from a poor outcome to death. The states and transitions, as well as a clinical control of the hazards for the transition through states, should improve the physician’s decision-making process. Since gender and age are difficult to control, early intervention by patients and the hospital may be critical in influencing functional outcomes.

**Supplementary Information:**

The online version contains supplementary material available at 10.1186/s12883-023-03444-8.

## Background

Stroke is one of the world’s major causes of death, morbidity, and long-term disability. It is the second-top cause of death in the world and the third-leading cause of Disability-Adjusted Life Years (DALYs) [1]. In 2017, there were 11.9 million incidents, 104.2 million recurring, 6.2 million fatal, and 132.1 million DALY-associated stroke cases. Between 1990 and 2017, the total number of people who developed, died, lived, or remained disabled as a result of a stroke nearly doubled. The bulk of stroke burden (80% of all incident strokes, 77% of all stroke survivors, 87% of all deaths from stroke, and 89 of all stroke-related DALYs) in 2017 was in low- to middle-income countries [2]. According to WHO data published in 2017, the age-adjusted death rate of stroke in Ethiopia is 89.82 per 100,000 of the population [3]. Approximately half of the stroke patients in the study area had unsatisfactory treatment outcomes [4].

A stroke occurs when the blood supply to the brain is interrupted or ruptured, resulting in the death of some brain cells owing to a lack of oxygen; strokes are also a leading cause of dementia and depression [5]. Some risk factors, such as high blood sugar levels, high blood pressure, and an irregular pulse, may contribute to functional disability [6]. Adults (over the age of 60) were more likely to have poor functional status after a stroke [7]. Post-acute care and rehabilitation are typically considered as a means of decreasing costs as healthcare systems adapt in response to reform attempts, despite their therapeutic impact and capacity to minimize the risk of downstream medical morbidity caused by reduced functional independence [8].

The Modified Rankin Scale (MRS) is used to evaluate functional ability in stroke patients, as the scale runs from 0 to 6, running from perfect health without symptoms to death [9]. However, such studies typically still analyze repeated measures of MRS (baseline; months 1, 3, 6, 9, and 12) to analyze thrombolysis and endovascular treatment effects on ordinal MRS with functional recovery but do not identify associations with mortality nor incorporate the treatment effect beyond one year [10]. MRS was studied at baseline, 3, 6, 9, 12, 15, 18, 21, 24, 27, 30, 33, and 36 months. The main objective of this study was to identify stroke-related functional outcomes and risk factors. It makes two contributions. First, by applying a Markov model, we infer the different states that affect stroke-related functional outcomes. Second, we identify states in which early intervention may be critical to influencing functional outcomes.

A large number of risk factors are measured in stroke disease studies, but it is often unclear whether all of them are relevant variables and whether the impact of these variables changes over time or remains constant with the rate of transition between various states of functional disability in stroke patients. The time to measure functional loss at various states and quantify the influence of key factors is limited. The overall and patient-specific mean length of functional recovery in various functional states was determined using a multistate model, and the impact of clinical factors on functional transitions was thoroughly investigated [11]. Modeling acute stroke therapy trial data because follow-up is frequently not scheduled at uniformly spaced intervals, but by putting in place the referral system for evaluation and management of stroke complications, stroke patients need and keep quarterly follow-up appointments [12]. A good functional outcome is defined as the absence of problems secondary to the stroke event, a poor functional outcome as the presence of complications, and a mortality outcome as the existence of complications. Good and poor outcomes were characterized as temporary states from which transition was permitted, and death state as an absorbing condition from which transition was not permitted. Gender, age of stroke patients, hypertension, diabetes mellitus, atrial fibrillation, types of stroke, and Glasgow coma scale score as covariates in the model via transition intensities, diabetes mellitus, and atrial fibrillation were risk factors for death.

## Methods

### The data

Retrospective data was acquired by reviewing the records of patients who received follow-up care at Felege Hiwot Referral Hospital between September 2019 and August 2021. The hospital has 500 formal beds, 11 wards, 39 clinical and nonclinical departments, and serves nearly 7 million people from the surrounding Amhara region of Ethiopia. The hospital treated 2262 stroke patients between September 2019 and August 2021. The Modified Rankin Scale is a frequently used scale for determining the degree of disability in the study area, and the physician (primary care physician, cardiologist, or neurologists) were documented as having rescheduled and completed a follow-up post-discharge period. The actual data collection took place between January 1/2022 and March 1/2022 and was based on a medical chart examination of stroke patients enrolled in the hospital’s Non-Communicable Disease Unit.

#### Study design

A retrospective cohort study was carried out to observe variables in a sample of stroke patients in order to predict outcomes. Three skilled clinical pharmacists extracted the information from the medical ward outpatient clinic center, according to the researcher’s agreement. Following data extraction, data entry, editing, coding, and organization were completed. R software version 4.2.2 was used to perform descriptive statistics. To perform inferential statistics, the R package MSM (version 1.6.8) was used.

#### Sampling design

**Fig. 1:**
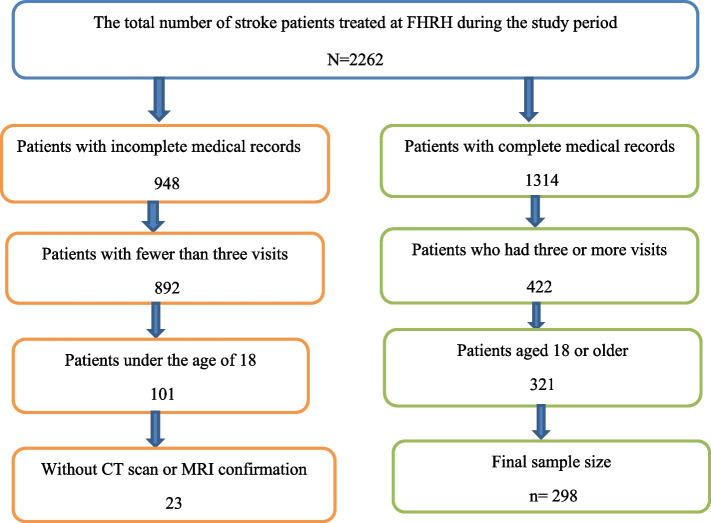
Shows the conceptual framework of the sampling procedure

Since there is no standard patient workup for stroke diagnosis, future guidelines will incorporate the most dependable time intervals for laboratory testing, such as CT scanning. Examinations were conducted at least once a week and, where possible, during periods of sickness deterioration. During the initiative of data gathered prospectively throughout the patient’s hospitalization and at particular follow-up intervals, four university hospitals could select a cohort of patients with a more severe stroke than community hospitals in America. Following hospital release, follow-up examinations were performed at 3, 6, 12, and 24-month intervals [13]. MRS improved by 1 point in 25.0% of patients from 3 months to 1 year after 1 year of progress in MRS was restricted. Although later recovery does occur, extending follow-up to one year would capture the majority of long-term stroke-related impairment, allowing for mortality follow-up of two years or more [14]. The MRS was used to evaluate both recovery from poor outcomes and the transition from poor outcomes to death at baseline in months 3, 6, 9, 12, 15, 18, 21, 24, 27, 30, 33, and 36. According to FHRH, there were 2262 stroke patients between September 2019 and August 2021. All adult (18 or older) stroke patients were considered for inclusion in the study. The study comprised 298 eligible adult (18 or older) stroke patients with relevant data on study variables at the beginning and following continuous recovery treatment within the first three years who were reviewed retrospectively by three clinical pharmacists based on their registration number (see Fig. 1: The conceptual framework of sampling procedure).

#### Inclusion and exclusion criteria

This study included medical data from stroke patients who had complete medical records, had at least three visits, were 18 or older, and had a CT scan or MRI confirmation between September 2019 and August 2021. Stroke patients with unfit medical records, those with less than three visits, those under the age of 18, and those without a CT scan or an MRI were also excluded from the study.

#### Operational definitions

*Functional disability* is the acquired difficulty in performing basic everyday tasks or more complex tasks needed for independent living.

*Outpatient care* is a medical service that does not require an overnight stay in a hospital.

*Atrial fibrillation* is an abnormal heartbeat characterized by rapid and irregular beating of the atrial chambers of the heart.

*Ischemic stroke* is a type of stroke that occurs when the blood supply to part of the brain is interrupted or reduced, preventing brain tissue from getting oxygen and nutrients.

*Hemorrhage stroke* is bleeding in or around the brain that interferes with the brain’s function and can be life-threatening.

*Glasgow coma scale* is a clinical scale used to reliably assess a person’s state of consciousness after a brain injury. It ranges from 3 (completely unresponsive) to 15 (responsive) and is classified as mild (13–15), moderate (9–12), and severe (3–8).

*Good outcome:* If the patient is without any complications secondary to the stroke attack.

*Poor outcome:* If patients had any complications.

#### Variables in the study

The MRS-based functional ability was used as a response variable in this investigation. It is used to measure the degree of functional ability in patients who have had a stroke, with 0 indicating no symptoms, 1 indicating no significant disability and the ability to carry out all usual activities despite some symptoms, 2 indicating slight disability and the ability to look after own affairs without assistance but unable to carry out all previous activities, 3 indicating moderate disability and the ability to walk unassisted, 4 for moderately severe disability, unable to attend to own bodily needs without help and unable to walk unassisted, 5 for severe disability, bedridden, incontinent, and 6 for dead [9]. The MRS is categorized as MRS < 3 as the good outcome and MRS ≥ 3 as the poor outcome [15–19]. In this study, adult (18 or older) stroke patients with MRS < 3 were considered as the good outcome (State 1), MRS ≥ 3 as the poor outcome (State 2) [15–19], and death (State 3) [9]. Independent variables were the gender of the patient (male, female), the patient’s age (18–59, 60 and above; there is clearly no conceptual justification for choosing one standard over another; hence, the choice is arbitrary), hypertension (Absent, Present), diabetes (Absent, Present), types of stroke (ischemic, hemorrhage), atrial fibrillation (Absent, Present), Glasgow coma scale (mild, moderate, severe) [20], heart disease (Absent, Present), residence (Rural, Urban), HIV/AIDS (Absent, Present), and stroke complications (Absent, Present). The ending category of the independent variable was deemed to be the treatment group, whereas the first was regarded to be the control group. The explanatory factors were selected from the literature and were all included in the models.

### Method of data analysis

A patient’s experience in a survival study can be modeled as a process with two states and one potential transition from an “alive” state to a “dead” state. However, in some studies, the “alive” condition is divided into two or more intermediate (transient) states, each of which corresponds to a different stage of the illness. Multi-state models can be used in such research to model patient movement between states. Issues of concern in these models include the estimation of progression rates, assessing the effects of individual risk variables, survival rates, and prognostic forecasting [21]. To represent movement between states of functional outcomes for stroke patients, we used a discrete-time multi-state Markov model with constant transition rates in this investigation. The death condition was absorbing because it does not allow for exits. Both good and poor outcomes were transient.

Longitudinal data were measurements of the disease process at various points in time. Although the underlying mechanism evolved continuously over time, the exact times at which state transitions happened were unknown. Kalbfleisch and Lawless (1985) developed the analysis of panel data under a Markov assumption in which transitional intensities control movement between disease states ${q}_{ij}\left(t,z\left(t\right)\right)$ with i, j = 1, 2, and 3 (the three possible states) and depend on time t and individual level or time-dependent explanatory factors at time t, denoted z(t) [22]. In our case, the ${q}_{ij }$form of a (3 × 3) Q matrix whose rows add up to zero, resulting in diagonal elements, is defined by ${q}_{ij}=-\sum _{i\ne j}{q}_{ij}$ Because it is not practical to transition from state III to either state I or state II, the transition intensity matrix is zero.

Marshall and Jones (1995) defined a new class of models by limiting the number of parameters, permitting all progressive or all regressive transitions to have the same regression coefficients, and introducing a new class of models, that is, $${q}_{ijl}\left(t\right)=\left\{\begin{array}{c}{q}_{ij}\left(0\right){ e}^{{\varvec{\beta }}_{p}{\varvec{z}}_{l}} j=i+1\\ {q}_{ij}\left(0\right){ e}^{{\varvec{\beta }}_{r}{\varvec{z}}_{l}} j=i-1\end{array}\right.$$ setting the regression parameters results in an even more restrictive model to ${ \varvec{\beta }}_{p}=-{\varvec{\beta }}_{r}=\varvec{\beta }$ [23]. Many covariates can be incorporated using the regression method [24].

Data are viewed as a sequence of observations in multi-state models ${x}_{i0},{x}_{i1},\dots ,{x}_{in}$ and at times ${t}_{i0},{t}_{i1},\dots ,{t}_{in}$ which is the product of $X\left(t\right)$process. In this process the amount of $1,\dots ,I$ states is $i=1,\dots ,N$ for each adult (18 or older) stroke patient, with covariate vectors ${z}_{l}$and model parameters *θ*, the log-likelihood under a Markov assumption can be expressed as [25] $L\left(\varvec{\theta }\right)=\sum _{i=1}^{N}\sum _{j=1}^{ni}\text{l}\text{o}\text{g}\left({p}_{{x}_{i\left(j-1\right)}{x}_{ij}}\right({t}_{i\left(j-1\right)},{t}_{ij};{z}_{l},\varvec{\theta }\left)\right)$ where, ${p}_{ij}\left({t}_{0},{t}_{1},\varvec{\theta }\right)=p(x\left({t}_{1}\right)=j|x\left({t}_{0}\right)=i;\varvec{\theta })$ and $(i,j)$ The transition chance can be calculated by solving the Kolmogrov Forward equation [26].

#### Model comparisons

For these models, Akaike’s Information Criterion (AIC) Akaike (1987) was used for model selection, $AIC=-2ln\left(likelihood\right)+2p$ where p is the number of parameters in the model and n is the number of subjects in the data or sample size.

#### Model diagnostics

The likelihood ratio test statistic was also used to assess the models’ time inhomogeneity [27]. Another common approach to assessing multi-state Markov models is to compare the observed prevalence of states with the expected prevalence under the model over time. To precisely compute the observed prevalence, all individuals should be observed at the same time. Gentleman et al. (1994) proposed a method for comparing observed and fitted data. This method is predicated on two assumptions. The first assumption is that an individual’s state at the time $t$was identical to their previous observation time. The second assumption is that the process starts at the same time for everyone [28].

## Results

Of the 2262 stroke patients eligible for inclusion in this study, 298 (13.2%) patients were included, and 1964 (86.8%) patients were excluded. Clinical and demographic variables were collected from adult (18 or older) stroke patients from September 2019 to August 2021 for the purpose of this study. The average and median mortality times for stroke patients were approximately 28 and 32 months after the initial diagnosis, respectively. This research included 298 adult (18 or older) stroke patients, with 162 (54.4%) being male and 136 being female. Most stroke patients (134 (47%)) were hypertensive, 96 (32.2%) had diabetes, 221 (74.2%) had ischemic stroke, 77 had hemorrhage stroke, 95 (31.9%) had atrial fibrillation disease, 72 (24.2%) had severe, 35 had moderate, and 191 had mild brain injuries. Table 1 depicts the state of functional outcomes for stroke patients at thirteen various observation time points throughout the month. At the time of their initial diagnosis, 93 stroke patients had a good outcome, while 205 stroke patients (69%) had a poor outcome.

**Table 1:** Functional outcomes of stroke patients at different time points in months at FHRH from September 2019 to August 2021

Visiting time in the month		State of functional outcome for stroke patients		
		Good state	Poor state	Death state
	Baseline	93	205	0
	3	123	174	0
	6	150	148	0
	9	163	114	20
	12	176	87	11
	15	172	76	12
	18	169	70	5
	21	157	63	9
	24	150	49	11
	27	141	43	9
	30	130	38	2
	33	122	28	4
	36	108	7	21
Total		1854	1102	104

### State transition between different possible states

According to Table 2, the research included 3060 longitudinal observations from 298 adult (18 or older) stroke patients over the first 36 months. (Three years). On two occasions, death states resulted from a good outcome, while 102 death states were the result of a poor outcome. In 167 instances, a good outcome was observed, followed by a poor outcome. An observation of a poor outcome was followed by an observation of a recovery to a good outcome 256 times. This finding validated the exclusion of some transitions based on the fact that transitioning from the absorbing (death) state to the transient state is impossible.

**Table 2:** The transition probability matrix was computed using data from stroke patients in various states

From To	Good	Poor	Death
Good	1505 (0.899)	167 (0.10)	2 (0.001)
Poor	256 (0.24)	730 (0.67)	102 (0.09)
Death	0 (0.00)	0 (0.00)	104 (1.00)

### The estimated transition intensities and hazard ratios for the covariates

The transition intensities between stages of functional outcomes in stroke patients were determined using a fitted multi-state Markov model with no variables. During the first three years of follow-up, the baseline transition intensities for functional loss from good to poor outcome were 10% and then for mortality were 9%, whereas the baseline transition intensities for functional recovery from poor to good outcome were 24%.

This model with covariates yielded a 95% confidence interval for the significant effects of stroke patients’ sex on the transition from good to poor outcome. The hazard of transitioning from a good to a poor outcome in women versus men was 1.54, with a 95% confidence interval ranging from 1.10 to 2.15. As a result, women are at a higher risk of stroke than men.

There was a 95% confidence interval for the significant effects of stroke patients’ age on the transition from good to poor outcome. The instantaneous risk of transitioning from a good to a poor outcome in stroke patients aged 60 or older versus those aged 18 to 59 was 1.73, with a 95% confidence interval ranging from 1.19 to 2.52. This implies that stroke patients over the age of 60 have higher morbidity and poor functional recovery after a stroke.

There was a 95% confidence interval that hypertension had a significant impact on stroke patients’ transition from good to poor outcome. The hazard of transitioning from a good to a poor outcome in stroke patient with hypertension versus those without hypertension was estimated to be 2.34, with a 95% confidence interval ranging from 1.55 to 3.53. This suggests that uncontrolled high blood pressure can impair stroke patients’ functional ability. High blood pressure damages vessels throughout the body, causing them to burst or clog more quickly.

Diabetes mellitus was found to have a significant impact on the transition from poor to good outcome and from poor outcome to death, with a 95% confidence interval. The instantaneous risk of transitioning from a poor to a good outcome in the stroke patient with diabetes mellitus versus those without diabetes mellitus was estimated to be 0.54, with a 95% confidence interval ranging from 0.35 to 0.85. The instantaneous risk of transitioning from poor outcome to death in the stroke patient with diabetes mellitus versus without diabetes mellitus was estimated to be 1.95, with a 95% confidence interval ranging from 1.10 to 3.46. This suggests that the effects of diabetes include damage to large and small blood vessels, which can lead to strokes, as well as problems with the nerves. Either the pancreas is not producing enough insulin or the body’s cells are not responding correctly to the insulin produced.

Atrial fibrillation was found to have a significant impact on the transition from good to poor outcome and from poor outcome to death, with a 95% confidence interval. The hazard of transitioning from a good to a poor outcome in stroke patient with atrial fibrillation versus without atrial fibrillation was estimated to be 2.74, with a 95% confidence interval ranging from 1.64 to 4.56. The hazard of transitioning from poor outcome to death in the stroke patient with atrial fibrillation versus without atrial fibrillation was estimated to be 3.39, with a 95% confidence interval ranging from 1.67 to 6.89. This suggests that blood clots are a risky complication of atrial fibrillation that can contribute to stroke. The abnormal heartbeat of atrial fibrillation may allow blood to collect in the upper chambers of the heart (atria) and create clots. If a blood clot in the left upper chamber (left atrium) of the heart escapes and travels to the brain, it may cause a stroke.

There was a 95% confidence interval that different types of stroke had significant impacts on the transition from good to poor outcome and poor to good outcome. The instantaneous risk of transitioning from a good to a poor outcome in patients with hemorrhage-type strokes versus those with ischemic-type strokes was estimated to be 1.52, with a 95% confidence interval ranging from 1.10 to 2.19. The instantaneous risk of transitioning from a poor to a good outcome in patients with hemorrhage-type strokes versus those with ischemic-type strokes was estimated to be 0.58, with a 95% confidence interval ranging from 0.42 to 0.80. This shows that various kinds of stroke can result in temporary or permanent disabilities, depending on how long the brain is without blood flow and which area of the brain is affected.

The Glasgow coma scale score was found to have a 95% confidence interval on the transition of stroke patients from poor to good outcomes. The hazard of transitioning from a poor to a good outcome in stroke patients with severe versus mild brain injury was found to be 0.77, with a 95% confidence interval ranging from 0.61 to 0.97. Because the brain controls movement and thought, a stroke can result in paralysis or death if the ability to think, move, and function is at risk.

### Estimated average time spent in a transient state

According to Table 3, the average amount of time spent in good outcome for female stroke patients was nearly 24 months; this implies that the lifetime risk of stroke is higher for women than men. The average amount of time spent on good and poor outcomes for stroke patients over the age of 60 was nearly 13 and 6 months, respectively. The average amount of time spent on good and poor outcomes for stroke patients with hypertension was nearly 11 and 7 months, respectively. The average length of time spent with good and poor outcomes for stroke patients with diabetes mellitus was nearly 10 and 6 months, respectively. The average length of time spent with good and poor outcomes for stroke patients with atrial fibrillation was nearly 9 and 7 months, respectively. The average amount of time spent on good and poor outcomes for hemorrhage stroke patients was nearly 16 and 7 months, respectively. For severely brain-injured stroke patients, the average amount of time spent on good and poor outcomes was nearly 14 and 6 months, respectively. This showed that, based on the risk factors and outcomes of stroke patients, the average time spent in transient states may be longer for one group than the other.

**Table 3:** Estimated average times spent on good and poor outcomes separately for each group of independent variables

Covariates	Covariate levels	Estimated average time spent in good state (months)	Estimated average time spent in poor state (months)
Gender of the patient	Male	27	8
	Female	24	8
Age of the patient	18-59	40	9
	60 or older	13	6
Hypertension	Absent	48	9
	Present	11	7
Diabetes Mellitus	Absent	36	7
	Present	10	6
Atrial Fibrillation	Absent	40	7
	Present	9	7
Types of stroke	Ischemic	28	10
	Hemorrhage	16	7
Glasgow Coma Scale Score	Mild	27	11
	Moderate	20	10
	Severe	14	6

### Results for model comparison

The maximum likelihood of the unknown parameters for the covariate model was better, with a lower AIC score of 2569.94, indicating that the chosen covariate model can predict future observations given that it best matches the data at hand.

### Model diagnostic check

**Fig. 2:**
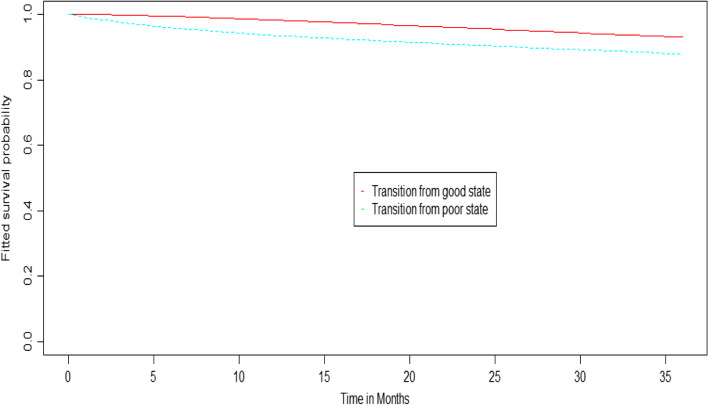
Plot of the expected probability of surviving in a certain state against time

In medical studies, the expected probability of survival versus time is used to calculate the proportion of patients who survive for a given period of time after treatment. The total survival function declined. Figure 2 depicts the expected probability of survival in a given state versus the average length of time spent for good outcomes (top) and poor outcomes (bottom). This finding suggests that stroke patients with a good outcome have survived longer than those with a poor outcome before dying. That is, as time progresses, the chances of adult (18 or older) stroke patients surviving decline.

**Fig. 3:**
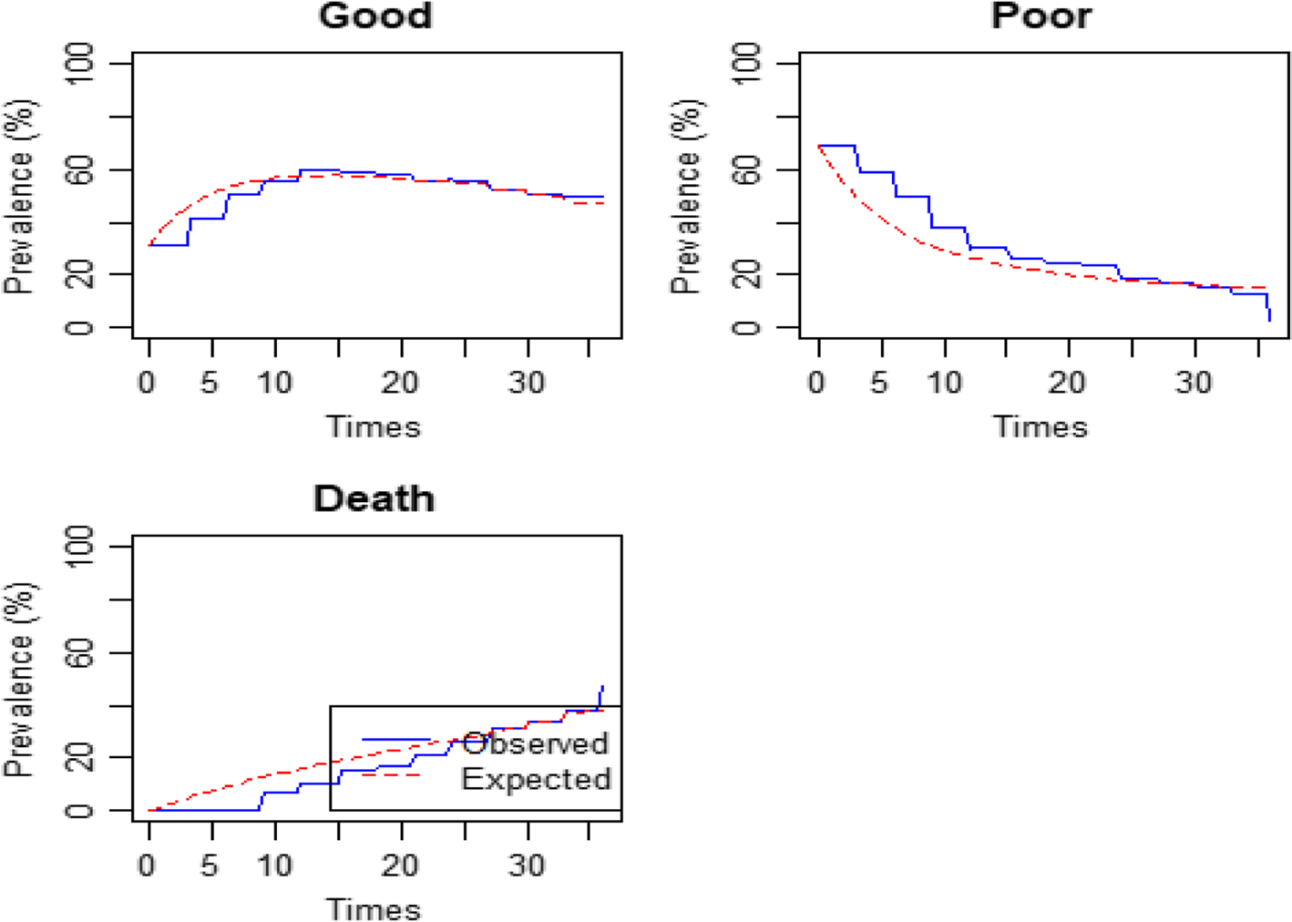
Prevalence plot of the fitted model with covariates

The prevalence plots approximate the goodness of fit of a multi-state model with variables. The actual prevalence is shown in solid lines, while the expected prevalence is shown in dashed lines. The model slightly overestimated the good outcome, underestimated the poor outcome, and overestimated death about 6, 12, and 21 months backward, as shown in Fig. 3. An omitted covariate could explain such differences. If the longitudinal data shows an increasing or decreasing pattern and the transition intensities are permitted to rely on covariates, the Markov property should be better fitted.

Using the likelihood ratio test, the null hypothesis of time inhomogeneity in the models was rejected (*p*-value = 0.000), and the alternative covariate model showed a significant improvement over the null model.

## Discussions

We validated the well-known findings of the experts, namely that the chances of adult (18 or older) stroke patients surviving decline over time. The likelihood of a poor outcome among stroke patients decreased as follow-up time (visit time) increased [29]. In contrast to Pan et al., who set the chance of worsening changes in good to poor outcome at zero [11], we observed that stroke patients with good outcomes lived longer than those with poor outcomes. The risk of shifting from a good to a poor outcome was 10%, and from a poor outcome to death was 9%. Other investigations indicated that poor functional status was more common in stroke patients with stroke durations of more than 12 months [7]. The impact of disability on survival is greater than that of numerous other well-known prognostic variables [19]. Furthermore, we found that the chance of shifting from a poor to a good outcome was 24%. This finding implies that interventions that promote an early shift toward lower MRS scores could minimize both long-term disability and mortality [14]. The baseline transition intensities for functional recovery from poor to moderate, and then to good, were calculated to be 0.27 and 0.14, respectively [11]. The gap resulted from a variation in how absorbing and exit states were characterized (i.e., death state instead of good outcome).

According to this study, female stroke patients were more likely to progress from a good to a poor outcome. Women were more likely to have poor functional status, which was associated with a poorer likelihood of post-stroke survival [7, 15, 30]. Several studies [31, 32] suggest that women with stroke had a greater death rate and a worse functional result than males. One plausible explanation is that women generally live longer than men; more women have strokes over their lifetimes. Some risk factors may also contribute to this situation, such as atrial fibrillation, hypertension, and diabetes mellitus, which are more common in women [33–35]. The gender of stroke patients, on the other hand, had no effect on the transition between functional levels [11].

A number of studies in this area have shown that as adults’ ages increase, so does their risk of death [36, 37]. Stroke patients’ age increased their likelihood of having impaired functional abilities [7, 15, 38]. In our analysis, stroke patients aged 60 or older were more likely to progress from a good to a poor outcome than those aged 18 to 59. The chance of a moderate to good functional status was significantly impacted by patients’ age; younger stroke patients recovered faster [11]. This is due to the older population’s reduced muscle strength, altered walking, poor balance, and use of specific and many drugs, which contribute to the overall high stroke incidence [30, 39].

The majority of other studies determined that hypertension was a significant risk factor for stroke [9, 40, 41]. This study showed that stroke patients with hypertension were more likely to experience a transition from a good to a poor prognosis. The results of this study suggest that controlling high blood pressure is critical for lowering the risk of stroke-related functional impairment. This is due to hypertension hardening atherosclerosis and causing blood vessel constriction, leading to stroke [6]. Hypertension has been recognized as a significant predictor of poor survival time and decreased functional outcome after stroke [19, 37].

Diabetes was found to be related to poorer outcomes following ischemic or hemorrhagic strokes in the majority of studies [17, 19, 30, 38, 41], including higher mortality, poorer neurological and functional results, longer hospital stays, higher readmission rates, and stroke recurrence. In this study, stroke patients with diabetes mellitus were more likely to progress from a poor outcome to death. High blood sugar levels accelerate the development of atherosclerosis (artery narrowing and hardening). This increases the risk of stroke significantly [6].

Most studies found that patients with atrial fibrillation had a higher risk of poor functional outcomes and death [15, 19, 42]. This study found that stroke patients with atrial fibrillation were more likely to have their health deteriorate from good to poor and then die. In response to irregular heartbeats, the heart does not contract as hard as it should. This can cause blood to pool in the heart and clot. When blood clots dislodge, they may travel to the brain and thus become stuck in a narrow brain artery, limiting blood flow and causing a stroke [6].

According to the findings of this study, stroke patients with hemorrhagic-type stroke were more likely to progress from a good to a poor outcome than those with ischemic-type stroke. Hemorrhagic stroke was associated with an increased risk of death [17, 43]. Some risk factors, such as high blood pressure and an irregular pulse, may contribute to this disability [6]. This is because treatment is dependent on whether the stroke was ischemic or hemorrhagic, how long it has been since symptoms began, and whether the patient has underlying medical conditions.

The study revealed that stroke patients with severe Glasgow coma scale scores were less likely than those with mild Glasgow coma scale scores to transition from poor to good outcomes. Other findings revealed that adult stroke patients with Glasgow coma scales 3–8 had a higher chance of death than patients with Glasgow coma scales 13–15 [44]. A lower admission Glasgow coma scale score and hemorrhagic stroke significantly raised the chance of mortality [17]. When this occurs, a portion of the brain no longer receives the blood and oxygen it requires, and it begins to die.

One of the study’s main limitations is the retrospective observational design. Because the study period was three years, the statistically significant likelihood of the shift from poor outcomes to mortality outcomes is likely to be overestimated. This may be due to the limitations of the multistate Markov model, including the difficulty in determining sample size and the computationally demanding nature of the model. A long-term follow-up could help identify significant transition intensities for functional recovery from a poor to a good outcome. More study is required to determine the effect of time-varying risk factors on transition intensities for functional recovery and mortality. Other limitations of this study included the exclusion of stroke patients without CT scan or MRI confirmation. And because stroke can happen to anyone, at any age, at any moment, everyone must be included, such as patients under the age of 20 and those who die at any age.

### Conclusion and recommendations

Women over 60 with hypertension, atrial fibrillation, and hemorrhagic stroke were more likely to progress from a good to a poor outcome. Diabetes and atrial fibrillation were also risk factors for progressing from a poor outcome to death.

The states and transitions, as well as a clinical control of the hazards for the transition through states, should improve the physician’s decision-making process. Since gender and age are difficult to control, early intervention by patients and the hospital may be critical in influencing functional outcomes. A future study should focus on stroke patients who did not have a CT scan or an MRI and patients of any age at any time.

### Supplementary Information

**Additional File 1: Figure 1.** Three state model for states of Functional Ability of Stroke patients.

## Data Availability

The datasets generated and analyzed during the current study are available upon reasonable request from the corresponding author.

## Abbreviations

AIC: Akaike’s Information Criterion
CT: Computerized Tomography
DALY: Disability-adjusted life year
FHRH: Felege Hiwot Referral Hospital
MRI: Magnetic Resonance Imaging
MRS: Modified Rankin Scale
MSM: Multi State Model
WHO: World Health Organization

## References

[1] World Health Organization. (2020). Global health estimates: life expectancy and leading causes of death and disability. Available from: https://www.who.int/data/gho/data/themes/mortality-and-global-health-estimates.

[2] Krishnamurthi RV, Ikeda T, Feigin VL (2020). Global, regional and country-specific burden of ischaemic Stroke, intracerebral haemorrhage and Subarachnoid Haemorrhage: a systematic analysis of the global burden of Disease study 2017. Neuroepidemiology.

[3] Alene M, Assemie MA, Yismaw L, Ketema DB (2020). Magnitude of risk factors and in-hospital mortality of Stroke in Ethiopia: a systematic review and meta-analysis. BMC Neurol.

[4] Kefale B, Ewunetei A, Molla M, Tegegne GT, Degu A (2020). Clinical pattern and predictors of Stroke treatment outcome among hospitalised patients who had a Stroke at Felege Hiwot comprehensive specialised hospital, northwest Ethiopia: a retrospective cross-sectional study. BMJ Open.

[5] Johnson W, Onuma O, Owolabi M, Sachdev S (2016). Stroke: a global response is needed. Bull World Health Organ.

[6] World Health Organization. Avoiding heart attacks and strokes: don't be a victim-protect yourself. World Health Organization. 2005.

[7] Zhou J, Liu F, Zhou M, Long J, Zha F, Chen M, Li J, Yang Q, Zhang Z, Wang Y (2022). Functional status and its related factors among Stroke survivors in rehabilitation departments of hospitals in Shenzhen, China: a cross-sectional study. BMC Neurol.

[8] Winstein CJ, Stein J, Arena R, Bates B, Cherney LR, Cramer SC, Deruyter F, Eng JJ, Fisher B, Harvey RL, Lang CE (2016). Guidelines for adult Stroke rehabilitation and recovery: a guideline for healthcare professionals from the American Heart Association/American Stroke Association. Stroke.

[9] Wilson JL, Hareendran A, Hendry A, Potter J, Bone I, Muir KW (2005). Reliability of the modified Rankin Scale across multiple raters: benefits of a structured interview. Stroke.

[10] Palesch YY, Yeatts SD, Tomsick TA, Foster LD, Demchuk AM, Khatri P, Hill MD, Jauch EC, Jovin TG, Yan B, von Kummer R, Palesch YY, Yeatts SD, Tomsick TA, Foster LD, Demchuk AM, Khatri P, Hill MD, Jauch EC, Jovin TG, Yan B, von Kummer R, Molina CA, Goyal M, Schonewille WJ, Mazighi M, Engelter ST, Anderson C, Spilker J, Carrozzella J, Ryckborst KJ, Janis LS, Simpson A, Simpson KN, Broderick JP (2015). Twelve-month clinical and quality-of-life outcomes in the Interventional Management of Stroke III trial. Stroke.

[11] Pan SL, Lien IN, Yen MF, Lee TK, Chen TH (2008). Dynamic aspect of functional recovery after Stroke using a multistate model. Arch Phys Med Rehabil.

[12] Cassarly C, Martin RH, Chimowitz M, Peña EA, Ramakrishnan V, Palesch YY (2017). Comparison of multistate Markov modeling with contemporary outcomes in a reanalysis of the NINDS tissue plasminogen activator for acute ischemic Stroke treatment trial. PLoS ONE.

[13] Kunitz SC, Gross CR, Heyman A, Kase CS, Mohr JP, Price TR, Wolf PA (1984). The pilot Stroke Data Bank: definition, design, and data. Stroke.

[14] Ganesh A, Luengo-Fernandez R, Wharton RM, Gutnikov SA, Silver LE, Mehta Z, Rothwell PM (2017). Oxford Vascular Study. Time course of evolution of disability and cause‐specific mortality after ischemic Stroke: implications for trial design. J Am Heart Association.

[15] Mengel A, Ulm L, Hotter B, Harms H, Piper SK, Grittner U, Montaner J, Meisel C, Meisel A, Hoffmann S (2019). Biomarkers of immune capacity, Infection and inflammation are associated with poor outcome and mortality after stroke-the PREDICT study. BMC Neurol.

[16] Bath P (2008). Calculation of sample size for Stroke trials assessing functional outcome: comparison of binary and ordinal approaches: the optimising analysis of Stroke trials (OAST) collaboration. Int J Stroke.

[17] Deljavan R, Farhoudi M, Sadeghi-Bazargani H (2018). Stroke in-hospital survival and its predictors: the first results from Tabriz Stroke Registry of Iran. Int J Gen Med.

[18] Panni P, Gory B, Xie Y, Consoli A, Desilles JP, Mazighi M, Labreuche J, Piotin M, Turjman F, Eker OF, Bracard S, Panni P, Gory B, Xie Yu, Consoli A, Desilles J-P, Mazighi M, Labreuche J, Piotin M, Turjman F, Eker OF, Bracard S, Anxionnat R, Richard S, Hossu G, Blanc R, Lapergue B, Redjem H, Escalard S, Ciccio G, Smajda S, Fahed R, Obadia M, Sabben C, Corabianu O, de Broucker T, Smadja D, Alamowitch S, Ille O, Manchon E, Garcia P-Y, Taylor G, Maacha MB, Bourdain F, Decroix J-P, Wang A, Evrard S, Tchikviladze M, Coskun O, Di Maria F, Rodesh G, Leguen M, Tisserand M, Pico F, Rakotoharinandrasana H, Tassan P, Poll R, Nighoghossian N, Labeyrie PE, Riva R, Derex L, Cho T-H, Mechtouff L, Claire Lukaszewicz A, Philippeau F, Cakmak S, Blanc-Lasserre K, Vallet A-E (2019). Acute Stroke with large ischemic core treated by thrombectomy: predictors of good outcome and mortality. Stroke.

[19] Eriksson M, Norrving B, Terént A, Stegmayr B (2008). Functional outcome 3 months after Stroke predicts long-term survival. Cerebrovasc Dis.

[20] Jain S, Iverson LM. Glasgow Coma Scale. In: StatPearls. Treasure Island (FL): StatPearls Publishing; 2022. 30020670

[21] Meira-Machado L, de Uña-Álvarez J, Cadarso-Suárez C, Andersen PK (2009). Multi-state models for the analysis of time-to-event data. Stat Methods Med Res.

[22] Kalbfleisch JD, Lawless JF (1985). The analysis of panel data under a Markov assumption. J Am Stat Assoc.

[23] Marshall G, Jones RH (1995). Multi-state models and diabetic retinopathy. Stat Med.

[24] Christodoulou G, Taylor GJ (2001). Using a continuous time hidden Markov process, with covariates, to model bed occupancy of people aged over 65 years. Health Care Manag Sci.

[25] Titman AC (2009). Computation of the asymptotic null distribution of goodness-of-fit tests for multi-state models. Lifetime Data Anal.

[26] Cox DR, Miller HD (1965). The theory of stochastic processes.

[27] Lawless JF, Nazeri Rad N (2015). Estimation and assessment of Markov multistate models with intermittent observations on individuals. Lifetime Data Anal.

[28] Gentleman RC, Lawless JF, Lindsey JC, Yan P (1994). Multi-state Markov models for analysing incomplete Disease history data with illustrations for HIV Disease. Stat Med.

[29] Synhaeve NE, Arntz RM, Maaijwee NA, Rutten-Jacobs LC, Schoonderwaldt HC, Dorresteijn LD, de Kort PL, van Dijk EJ, de Leeuw FE (2014). Poor long-term functional outcome after Stroke among adults aged 18 to 50 years: Follow-Up of transient ischemic Attack and Stroke patients and unelucidated risk factor evaluation (FUTURE) study. Stroke.

[30] Radisauskas R, Tamosiunas A, Kranciukaite-Butylkiniene D, Milinaviciene E, Malinauskiene V, Bernotiene G, Luksiene D, Virviciute D, Rastenyte D (2019). Long-term survival after Stroke in Lithuania: data from Kaunas population-based Stroke registry. PLoS ONE.

[31] Rexrode KM, Madsen TE, Yu AY, Carcel C, Lichtman JH, Miller EC (2022). The impact of sex and gender on Stroke. Circul Res.

[32] Benjamin EJ, Muntner P, Alonso A, Bittencourt MS, Callaway CW, Carson AP, Chamberlain AM, Chang AR, Cheng S, Das SR, Delling FN (2019). Heart Disease and Stroke statistics—2019 update: a report from the American Heart Association. Circulation.

[33] Maeda K, Toyoda K, Minematsu K, Kobayashi S, Japan Standard Stroke Registry Study Group (2013). Effects of sex difference on clinical features of acute ischemic Stroke in Japan. J Stroke Cerebrovasc Dis.

[34] Niewada M, Kobayashi A, Sandercock PA, Kamiński B, Członkowska A (2005). Influence of gender on baseline features and clinical outcomes among 17,370 patients with confirmed ischaemic Stroke in the international Stroke trial. Neuroepidemiology.

[35] Peters SA, Carcel C, Millett ER, Woodward M (2020). Sex differences in the association between major risk factors and the risk of Stroke in the UK Biobank cohort study. Neurology.

[36] Sarfo FS, Akassi J, Kyem G, Adamu S, Awuah D, Kantanka OS, Ovbiagele B (2018). Long-term outcomes of Stroke in a Ghanaian outpatient clinic. J Stroke Cerebrovasc Dis.

[37] Yao M, Ni J, Zhou L, Peng B, Zhu Y, Cui L, SMART investigators (2016). Elevated fasting blood glucose is predictive of poor outcome in non-diabetic Stroke patients: a sub-group analysis of SMART. PLoS ONE.

[38] Kuwashiro T, Sugimori H, Ago T, Kuroda J, Kamouchi M, Kitazono T (2013). The impact of predisposing factors on long-term outcome after Stroke in diabetic patients: the F ukuoka S troke R egistry. Eur J Neurol.

[39] Someeh N, Shamshirgaran SM, Farzipoor F, Asghari-Jafarabadi M (2020). The moderating role of underlying predictors of survival in patients with brain Stroke: a statistical modeling. Sci Rep.

[40] Erkabu SG, Agedie Y, Mihretu DD, Semere A, Alemu YM (2018). Ischemic and hemorrhagic Stroke in Bahir Dar, Ethiopia: a retrospective hospital-based study. J Stroke Cerebrovasc Dis.

[41] Gedefa B, Menna T, Berhe T, Abera H (2017). Assessment of risk factors and treatment outcome of Stroke admissions at St. Paul’s teaching hospital, addis ababa, Ethiopia. J Neurol Neurophysiol.

[42] Song TJ, Baek IY, Woo HG, Kim YJ, Chang Y, Kim BJ, Heo SH, Jung JM, Oh K, Kim CK, Yu S, Song T-J, Baek I-Y, Woo HG, Kim Y-J, Chang Y, Kim BJ, Heo SH, Jung J-M, Oh K, Kim CK, Yu S, Park KY, Kim J-M, Park J-H, Choi JC, Park M-S, Kim J-T, Choi K-H, Hwang Y-H, Chung J-W, Bang OY, Kim G-M, Seo W-K (2019). Characteristics and factors for short-term functional outcome in Stroke patients with atrial fibrillation, nationwide retrospective cohort study. Front Neurol.

[43] Labodi LD, Kadri C, Valentin YN, Christian N, Jean KB (2017). Intra-hospital mortality of Stroke and its predictive factors in a reference hospital in Ouagadougou, Burkina Faso. Brain.

[44] Hagos Gufue Z, Gizaw NF, Ayele W, Yifru YM, Hailu NA, Welesemayat ET, Tsegay EW, Atsbaha AH, Gebru HT (2020). Survival of Stroke patients according to Hypertension status in Northern Ethiopia: seven years retrospective cohort study. Vascular Health Risk Manag.

