# Supplementary material for: Risk factors for stroke-related functional disability and mortality at Felege Hiwot Referral Hospital, Ethiopia
Source: BMC Neurol. 2023 Oct 31;23:393. doi: 10.1186/s12883-023-03444-8 (PMC10617073; doi:10.1186/s12883-023-03444-8)
Supplement: Supplementary file 1 — Additional File 1: Figure 1. Three state model for states of Functional Ability of Stroke patients. [file 12883_2023_3444_MOESM1_ESM.docx]

### Multi State Model

Multi state models are an approach to analyzing categorical longitudinal data. A multi-state process is a stochastic process $(X\left( t \right), t\in T)$ with a finite state space
$S=\left\{ 1, \ldots, N \right\}$. Here, $T=\left[ 0,\tau\right], \tau<\infty$ is a time interval and the value of the process
at time *t* is the state occupied at that time. With the evolution of the process over time, a history $H_{t}$ will be generated consisting of the observation of the process over the interval [0*, t*), such as the states previously visited, times of transitions, etc. The multi-state process is fully characterized through transition probabilities between states *i* and *j*,

$p_{ij}\left( s,t \right)=P(X\left( t \right)=j|X\left( s \right)=i,H_{s-})$ $for i,j\in S, s,t\in T, s\leq t$ *……………* (1)

or through transition intensities

$\alpha_{ij}\left( t \right)=\lim_{\Delta t\to0} \frac{p_{ij}\left( t,t+\Delta t \right)}{\Delta t}$….………………………………….….……. (2)

representing the instantaneous hazard of progression to state *j* conditionally on occupying state *i*, and that we shall assume exist. Some transition intensities maybe 0 for all *t*. A state $i\in S$is exiting state if for all$t\in T, j\in S, j\neq i, \alpha_{ij}\left( t \right)=0;$otherwise i is transient. The state probabilities $\pi_{i}\left( t \right)=Prob\left( X\left( t \right)=i \right)$are given by:

$\pi_{i}\left( t \right)=\sum_{j\in S} \pi^{j}(0)P_{ji}\left( 0,t \right)$…………………………………………...…….. (3)

Notice that both $p_{ji}\left( .,. \right)$ and thereby the $\alpha_{ij}\left( . \right)$ depend on both the probability measure and on the history, though this dependence has been suppressed in the notation. If
$\alpha_{ij}\left( t \right)$ only depends on the history via the state $i=X\left( t \right)$ occupied at *t* then the process is
Markovian. Sometimes one is interested in considering an extended history which also
includes observed covariates (Andersen and Keiding, 2002).

The states should represent the status of a stroke patients. Transitions from one state to another are referred to as “events”. They should reflect distinct changes in the functional ability of stroke patient conditions. The times of transitions are referred to as the “event times”. The direction of the arrows should be determined by the underlying nature of the event process. The arrows indicate which instantaneous state-to-state transitions are possible.

| **State 2**  Poor Functional Ability  **State 1**  Good Functional Ability  **State 3**  Death state |
| --- |

Figure 1 Three state model for states of Functional Ability of Stroke patients
